# The relationship between perceptions of school climate and internet gaming disorder of teenage students: A moderated mediation model

**DOI:** 10.3389/fpsyg.2022.917872

**Published:** 2022-08-08

**Authors:** Mengrou Zhang, Wenhua Zhang, Yunhe Li, Xiangcai He, Feng Chen, Ying Guo

**Affiliations:** ^1^Zhoukou Vocational and Technical College, Zhoukou, China; ^2^School of Psychology, Guizhou Normal University, Guiyang, China; ^3^School of Physical Education, Zhoukou Normal University, Zhoukou, China; ^4^School of Psychology, Sichuan Normal University, Chengdu, China

**Keywords:** teenager students, school climate, maladaptive cognition, belief in a just world, internet gaming disorder

## Abstract

**Background:**

In recent years, teenage students’ internet gaming disorder has raised widespread concern in the society. The current study aims to explore how perceptions of school climate, maladaptive cognition, and belief in a just world impact teenage students’ internet gaming disorder and seek the suitable intervention to prevent teenage students’ internet gaming disorder tendency.

**Methods:**

A total of 1,164 teenage students (age: 19.62 ± 3.14 years) were evaluated using the Perceived School Climate Scale (PSCS), Maladaptive Cognition Scale (MCS), Belief in a Just World Scale (BJWS), and Internet Gaming Disorder Scale (IGDS).

**Results:**

(1) Perceptions of school climate could predict teenage students’ internet gaming disorder [β = –0.15, *p* < 0.001, 95% CI = (–0.20, –0.11)]. (2) Teenage students’ maladaptive cognition exerts a partial mediating effect between their perceptions of school climate and internet gaming disorder [ab = –0.13, boot *SE* = 0.01, 95% CI = (–0.16, –0.11)]. (3) The direct effect of perceptions of school climate on internet gaming disorder [β = –0.07, *p* < 0.01, *95% CI* = (–0.11, –0.02)], the first half of the mediation model “perceptions of school climate → maladaptive cognition” [β = –0.08, *p* < 0.01, 95% *CI* = (–0.12, –0.03)], and the second half of the mediation model “maladaptive cognition → internet gaming disorder” [β = 0.10, *p* < 0.001, 95% CI = (0.06, 0.14)] are all moderated reciprocally by teenage students’ belief in a just world.

**Conclusion:**

Schools should create a good climate and shape belief in a just world to reduce the maladaptive cognition of teenagers, to effectively prevent teenage students’ internet gaming disorder tendency.

## Introduction

In the mobile internet era, “digital survival” without leaving home brings great convenience to people’s life. The internet game was therewith born. Up to December 2020, the number of internet game users in China was 518 million, accounting for 52.4 percent of internet users (China Internet Network Information Center [CNNIC], 2021). Internet gaming is beneficial to individuals’ development (Przybylski, 2014; Lorentz et al., 2015). However, it should be moderate; otherwise, the reverse result will occur. Teenage years form the critical period for the formation of outlook on the world, values, and life. Teens develop their own set of guiding principles that regulate their behavior. Some teenage students may feel confused without the guidance of clear life goals during this time. Thus, internet games with the thrilling competition, dangerous plot, and the fantastic wonderland excitement as a bright light penetrating through dark clouds under great pressure captivates or even enslaves them. What follows is loss of the interest in learning, neglecting their studies or even staging a tragic life (Yesilyurt, 2020; Ohayon and Roberts, 2021). Internet gaming disorder was listed as a mental illness and integrated into the medical system by the World Health Organization in June 2018 (World Health Organization [WHO], 2019). At present, academia has spread research on internet gaming disorder and targeted therapy accurately (Pasquale et al., 2020; Wang et al., 2022), but most of them study the treatment methods based on the perspective of pathological mechanism, such as pharmacotherapy and reality psychotherapy (Park et al., 2017; Zhang and Ho, 2017). Analyzing from the view of the interfering factors and mechanisms of teenage students’ internet gaming disorder to explore preventive measures, we can believe it is a promising way of multiplying the effectiveness of our own efforts.

## Related background and hypothesis

### The relationship between perceptions of school climate and internet gaming disorder

Internet gaming disorder is a kind of game behavior with impaired physical, psychological, and social functions caused by overreliance on internet (Yu et al., 2015). According to the behavioral dynamics theory, actions arise from one’s need, and the need is a state of tension caused by some sort of deficiency. In the final analysis, it is the reflection of individual survival and developmental requirements. The tension would drive individuals’ behavior to seek methods to satisfy themself. As individuals in social life, the generation of needs and the choice of ways to satisfy themself will be affected by the social environment, such as experience, hobbies, values, and cultural customs (Li and Yao, 2001). According to the ecological systems theory put forward by Bronfenbrenner, the environment systems that individuals contact from inside to outside are the microsystem, intermediate system, outer system, and macrosystem, and the microenvironment, which has a long-term latent impact on individuals, is the closest and most direct factor to individuals’ behavior (Shaffer and Kipp, 2009). The school is the main battle position for teenage students, and their relationships with teachers and classmates are their main social relations, and those relationships playing roles in the teenage students’ microenvironment affect their development of physical and mental health. Based on this situation, researchers have included perceptions of school climate into the research category of the influence factors of teenage students’ internet gaming disorder (Zhu et al., 2015; Chang and Kim, 2019).

Perceptions of school climate refers to the characteristics of school climate that is experienced by members and has an impact on their behavior continuously as well, including the influence of teachers on individuals, the influence of classmates on individuals, and the autonomization from individuals (Jia et al., 2009). According to the stage-environment fit theory, the fit of learning resources and individual needs can promote the development of teenagers, and it emphasizes the important role of school climate in individuals’ growth (Kumar, 2002). Correlative empirical studies indicated that it could affect the positive emotions and behaviors of teenage students by the intervention of teacher support, classmate support, and independent opportunity (Tian et al., 2013; Yu et al., 2015). The satisfaction of social needs can promote the positive growth of individuals. On the contrary, when the social needs cannot be satisfied, the tension of the body will drive the individuals to seek compensation for unmet social needs elsewhere (Alfred, 2009), such as turning into internet games to seek for support, relieving the tension, and remolding the social support system in the virtual world gradually, indulging in it and not being able to extricate oneself from it (Zhang et al., 2021). Based on the theoretical and empirical grounds, we hypothesize that perceptions of school climate can predict teenage students’ internet gaming disorder.

### The mediating role of maladaptive cognition

Correlative studies indicated that individuals’ internet gaming disorder was mainly affected by the external environment and the internal drive (Xu et al., 2012; Kuss and Olatz, 2016). According to the cognitive–behavioral model of pathological internet use (PIU) put forward by Davis, cognition is the forerunner of behavior; meanwhile, irrational thoughts and attitudes toward the internet world—called maladaptive cognition—play an important role in the formation of internet gaming disorder. The formation of PIU is the combined effect of many factors, including not only psychopathological sources such as stressful life events and personal compatibility but also maladaptive cognition. These influencing factors were divided into two categories in the model: psychopathological sources, which act as the distal factor of PIU, and maladaptive cognition, which acts as the proximal factor of PIU; the distal factor works through the proximal factor (Davis, 2001; Mai et al., 2012). Meanwhile, correlative empirical studies also indicated that maladaptive cognition played a mediating role in the relationship between stress and internet gaming disorder (Yi et al., 2016), the relationship between shyness and internet gaming disorder (Tian et al., 2015), and the relationship between relative deprivation and internet gaming disorder (Ding et al., 2018).

The main source of individuals’ maladaptive cognition is the irrational cognition toward themselves and the irrational cognition toward the world (Mai et al., 2012). Previous studies show that a good social support system would contribute to the formation of individuals’ positive cognition such as a sense of belonging and wellbeing (Borkar, 2016; Gabriella et al., 2020; Renick and Reich, 2020; Zhou, 2020). On the contrary, individuals with childhood trauma and social exclusion are more likely to hold negative cognition and emotional experience toward the world and themselves (Choi et al., 2020; Angelakis and Gooding, 2022), which promotes individuals to explore a new path to escape from the old environment, where they can establish their new identity and satisfy themselves. At present, the characteristics of internet games’ anonymity and virtualization make them seem suitable for their needs perfectly and easily attract the individuals to produce maladaptive cognition (Hsu et al., 2009; Callan et al., 2015). According to the social learning theory, individuals live in society, and their behavior is closely related to their living environment and their cognition, which emphasizes the interaction of the environment, cognition, and behavior in the process of social learning (Ling, 2009). Therefore, as an environmental factor that has a significant impact on teenage students, perceptions of school climate may also affect internet game disorder through the impact of maladaptive cognition.

### The moderating role of belief in a just world

As an environment factor, perceptions of school climate affects individuals’ cognition and behavior. However, for individuals who have the same perception of school climate, the cognition and behavior are not the same. It shows that the interior resources of individuals may play a moderating role among the factors. Belief in a just world belongs to the interior resources of individuals, which is a belief in the justice of the world, including both the belief in the justice of the world to oneself and the belief in the justice of the world to others (Lerner and Miller, 1978). A plethora of studies suggested that the belief in a just world could promote individuals’ mental health (Jiang et al., 2016; Bartholomaeus and Strelan, 2021). With regard to the mechanism of the relationship, the mostly supported assumption is that the belief in a just world is a kind of personal resource. The more powerful interior resources one has, the better one can deal with challenges in life (Christandl, 2013). According to this view, individuals with a high level of justice in the world can adjust the impact on behavior by their powerful interior resources when facing the lower perception of school climate. This also explains the phenomenon that individuals with the same perception of school climate have different behaviors. So, we put the variable of belief in a just world into the path of perceptions of school climate and internet gaming disorder to test the moderating effect. In addition to the moderating effect as personal resources, the belief in a just world also affects individuals’ cognition. Correlative empirical studies indicated that individuals with stronger belief in a just world tend to solve problems with positive ideas and attitude more possibly, and their prosocial behaviors were more than others’ (Sutton and Winnard, 2007; Kaliuzhna, 2020). Thus, we put the variable of belief in a just world into the path of perceptions of school climate and maladaptive cognition to test the moderating effect. In the face of irrational cognition, the reconstruction of justice is also the function of the belief in a just world. On the action mechanism of the relationship, four strategies have been found at present: the first one was the rational strategy of reconstructing justice at the realistic level, the second one was the irrational strategy of reconstructing justice at the cognitive level, the third one was the protective strategy of placing hopes on future, and the fourth was the defensive strategy of cynicism (Lerner, 1980). After the cognition formed, as an internal resource of the individuals, which strategies will be chosen by the belief in a just world to act on behavior? We put the variable of belief in a just world into the path of maladaptive cognition and internet gaming disorder to test the moderating effect.

### The present study

**FIGURE 1:**
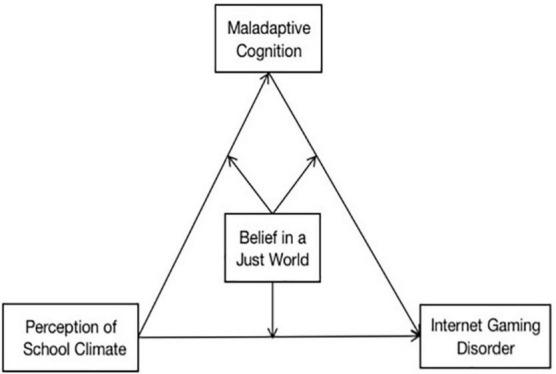
The conceptual diagram of moderated mediation model based on research hypothesis.

Relevant research studies have attained great achievements in the identification and diagnosis of internet game disorder. On these bases, the present study aims to explore the intervention measures through the research on the influencing factors and the mechanism of teenage students’ internet game disorder under the perspective of positive psychology so as to enrich the prevention and intervention system of teenage students’ internet game disorder. Based on previous theories and empirical studies, we put forward three hypotheses and constructed a moderated mediation model (Figure 1). The hypotheses are as follows:

H1: Perceptions of school climate can predict teenage students’ internet gaming disorder.

H2: Teenage students’ maladaptive cognition exerted a mediating effect between their perceptions of school climate and internet gaming disorder.

H3: The relationship between maladaptive cognition and internet gaming disorder was moderated reciprocally by teenage students’ belief in a just world.

## Materials and methods

### Participants and procedure

The survey was conducted from 16 February to 2 March 2022 in China. Participants were required to finish an online questionnaire *via* social media (QQ, Tencent). According to the age definition of teenagers (Zhang, 2002), 1,200 teenage students aged 14–25 years were selected as participants by convenient sampling from 31 public schools of central China, and the unified guidelines for group testing was used in the survey. Due to some reasons such as failure to submit the answer sheet, the final sample consisted of 1,164 participants by removing the uniform answers in all items. The number of male participants were 628 (age = 19.60 years, *SD* = 3.14), and the number of female participants were 536 (age = 19.64 years, *SD* = 3.13). Only child accounted for 38.9%, and non-only child accounted for 61.1%. In terms of the place of residence, 43.6% of teenage students were from cities, and 56.4% of teenage students were from countryside. The descriptive statistical characteristics of the sample are given in Table 1. All participants signed an electronic informed consent prior to their participation, and the consent of participants who were younger than 18 years were provided by the participants’ legal guardian/next of kin. They were informed that they could withdraw from the survey at any time if they would. All procedures performed in this study involving human participants were in accordance with the 1964 Helsinki Declaration and its later amendments or comparable ethical standards.

**TABLE 1:** Sociodemographic characteristics (*N* = 1,164).

Variables	Group	*N*	%
Gender	Male	628	54.00
	Female	536	46.00
Age	14–18 years	454	39.01
	18–25 years	710	60.99
Only child	Yes	453	38.90
	No	711	61.10
Place of residence	City	508	43.60
	Country side	656	56.40

### Measures

#### Perceptions of school climate

The Perceived School Climate Scale (PSCS) compiled by Jia was used to assess the level of perceived school climate in teenagers, and it comprised 24 items, which were divided into three dimensions: teacher support, classmate support, and independent opportunity (Jia et al., 2009). The measure uses a four-point scale scored form 1 (never) to 4 (always). The higher total scores indicate the better perception of school climate. Cronbach’s α-values of the three subscales among Chinese sample were 0.81 (teacher support), 0.86 (classmate support), and 0.82 (independent opportunity), and the reliability and validity of the scale were good (Liu et al., 2020; Zhai et al., 2020). Cronbach’s α was 0.91, and Cronbach’s α-values of the three subscales were 0.87 (teacher support), 0.88 (classmate support), and 0.77 (independent opportunity) in this study.

#### Maladaptive cognition

The Maladaptive Cognition Scale (MCS) compiled by Mai was used to assess the cognitive situation of internet, and it comprised 12 items, which were divided into three dimensions: social convenience, escape pressure, and self-realization in the scale (Mai et al., 2012). A five-point scale scored form 1 (strongly disagree) to 5 (strongly agree) was used to measure the different levels in the scale. The higher the total scores, the stronger the maladaptive cognition of internet of individuals. Cronbach’s α of the scale was 0.81, and Cronbach’s α-values of the three subscales were 0.66 (social convenience), 0.67 (escape pressure), and 0.74 (self-realization). In this study, Cronbach’s α of the scale was 0.94, and Cronbach’s α-values of the three subscales were 0.74 (social convenience), 0.88 (escape pressure), and 0.89 (self-realization).

#### Belief in a just world

The Belief in a Just World Scale (BJWS) compiled by Dalbert was usually used to assess the level of individuals’ belief in a just world. Its 13 items were divided into two dimensions: the general belief in a just world toward others and the personal belief in a just world toward oneself in the scale (Dalbert, 1999). The measure uses a six-point scale scored form 1 (strongly disagree) to 6 (strongly agree). The higher the total scores, the stronger the belief in a just world of individuals. Cronbach’s α of the scale was 0.86, and Cronbach’s α-values of the two subscales were 0.79 (general belief in a just world) and 0.85 (personal belief in a just world). Previous studies have confirmed that the reliability and validity of the scale were good (Wang et al., 2019; Tatsi and Panagiotopoulou, 2021). In this study, Cronbach’s α of the scale was 0.91, and Cronbach’s α-values of the two subscales were 0.87 (general belief in a just world) and 0.86 (personal belief in a just world).

#### Internet gaming disorder

The Different Types of Internet Addiction Scale compiled by Zhou was used to assess the differences among types of addiction (Zhou and Yang, 2006). The scale was formed by internet gaming disorder, cyber-relational addiction, and information overload. As a subscale, the Internet Gaming Disorder Scale (IGDS) consisted of eight items that reflected the infatuation with various internet games and the negative emotions caused by indulging in games. The IGDS could directly reflect the situation of internet gaming disorder, and the reliability and validity of the scale were confirmed well by previous studies (Zhang et al., 2021). So the IGDS was selected in this study. The measure uses a five-point scale scored form 1 (strongly disagree) to 5 (strongly agree). The higher the total scores, the higher the level of internet gaming disorder of individuals. Cronbach’s α of the scale was 0.88, and in this study, Cronbach’s α of the scale was 0.93.

#### Data analysis

SPSS 20.0 was used for data processing in this study. First, the factor analysis was used to test common method biases. Second, descriptive statistics were used to show the current situation of teenage students on various variables and Pearson correlations were used to reflect the relationship among the variables. Third, Model 4 of the PROCESS macro for SPSS was used to test the mediating effect of maladaptive cognition. Finally, Model 59 of the PROCESS macro for SPSS was used to test the moderating effects of belief in a just world among three paths of the mediation model. During the study, the bootstrap method (5,000 bootstrap samples) with 95% confidence interval (CI) was used to check the significance of the effects. In order to avoid the multi-pertinence, all observation variables were z-score-standardized before the analyses of Model 4 and Model 59. Considering that gender and age might influence the internet gaming disorder according to previous studies (Su et al., 2020; Lin et al., 2021), we regarded gender and age as control variables in model testing.

## Results

### Common method bias test

Since the data were collected by self-report questionnaires, they might have been affected by the systematical error caused by any factors such as data source and measurement environment (Zhou and Long, 2004). Although the control measures such as anonymous response have been adopted in the process of measurement, in order to improve the preciseness of the study, the method of Harman single factor was used to test the common method biases.

All items of scales were conducted by exploratory factor analysis, and the result indicated that nine factors with eigenvalues > 1 were extracted. The variance interpretation rate of the first common factor was 29.09%, which was less than the critical value of 40%. There was no case where only one factor was extracted or the variance interpretation rate of a factor was too large. Therefore, this study was not significantly affected by the common method biases.

### Comparison of study variables on sociodemographic characteristics

To explore the differences of perceptions of school climate, maladaptive cognition, belief in a just world, and internet gaming disorder among different types of teenage students, sociodemographic characteristics were taken as the independent variable, and the study variables were taken as the dependent variable. As shown in Table 2, the *t*-test showed that the factor of gender had significant effects on the perception of school climate (*t* = –9.25, *p* < 0.001), maladaptive cognition (*t* = 8.05, *p* < 0.001), belief in a just world (*t* = –6.48, *p* < 0.001), and internet gaming disorder (*t* = 11.04, *p* < 0.001). The perception reports about school climate and belief in a just world of female participants were higher than those in male participants, while the maladaptive cognition and the tendency of internet gaming disorder in female participants were lower than those in male participants. The factor of place of residence had a significant effect on the perception of school climate (*t* = 2.01, *p* < 0.05), and the perception reports in city teenagers were higher than those in countryside teenagers.

**TABLE 2:** Comparison of study variables on sociodemographic characteristics.

Variables	PSC		MC		BJW		IGD	
	M ± *SD*	*T*	M ± *SD*	*T*	M ± *SD*	*T*	M ± *SD*	*T*
**Gender**								
Male	2.82 ± 0.40	–9.25***	2.54 ± 0.73	8.05***	3.81 ± 0.86	–6.48***	2.55 ± 0.72	11.04***
Female	3.04 ± 0.44		2.17 ± 0.85		4.12 ± 0.76		2.02 ± 0.89	
**Age**								
14–18 years	2.93 ± 0.43	0.33	2.39 ± 0.81	0.50	3.94 ± 0.85	–0.14	2.32 ± 0.83	0.35
19–25 years	2.92 ± 0.43		2.36 ± 0.81		3.95 ± 0.82		2.30 ± 0.85	
**Only child**								
Yes	2.94 ± 0.43	1.07	2.39 ± 0.83	0.45	3.97 ± 0.82	0.55	2.28 ± 0.84	–0.76
No	2.91 ± 0.43		2.36 ± 0.79		3.94 ± 0.84		2.32 ± 0.84	
**Place of residence**								
City	2.95 ± 0.43	2.01*	2.38 ± 0.77	0.41	3.98 ± 0.80	1.11	2.33 ± 0.84	0.82
Countryside	2.90 ± 0.43		2.36 ± 0.83		3.93 ± 0.86		2.29 ± 0.85	

SD, standard deviation; PSC, perceptions of school climate; MC, maladaptive cognition; BJW, belief in a just world; IGD, internet gaming disorder; N = 1,164.

*P < 0.05; ***P < 0.001.

### Descriptive statistics and correlation analysis

The results of descriptive statistics and correlation analysis among variables of gender, age, only child, place of residence, perceptions of school climate, maladaptive cognition, belief in a just world, and internet gaming disorder on 1,164 teenage students are shown in Table 3.

**TABLE 3:** Descriptive statistics and intercorrelations between variables.

Variables	M ± *SD*	1	2	3	4	5	6	7	8
1. Gender	0.54 ± 0.50	–							
2. Age	19.62 ± 3.14	–0.01	–						
3. Only child	0.61 ± 0.49	0.03	0.04	–					
4. Place of residence	0.56 ± 0.50	–0.01	0.01	0.59***	–				
5. PSC	2.92 ± 0.43	–0.26***	–0.01	–0.03	–0.06*	–			
6. MC	2.37 ± 0.81	0.23***	–0.03	–0.01	–0.01	–0.41***	–		
7. BJW	3.95 ± 0.83	–0.19***	0.03	–0.02	–0.03	0.55***	–0.33***	–	
8. IGD	2.31 ± 0.84	0.31***	–0.04	0.02	–0.02	–0.41***	0.65***	–0.37***	–

SD, standard deviation; PSC, perceptions of school climate; MC, maladaptive cognition; BJW, belief in a just world; IGD, internet gaming disorder; gender is a dummy variable, 1, male, 0, female; only child is a dummy variable, 1, not, 0, yes; place of residence is a dummy variable, 1, countryside, 0, city; N = 1,164.

*P < 0.05; ***P < 0.001.

It could be seen from Table 3 that a significant negative correlation existed between perceptions of school climate and maladaptive cognition [*r* = –0.41, 95% CI = (–0.46, –0.36)], a significant positive correlation existed between perceptions of school climate and belief in a just world [*r* = 0.55, 95% CI = (0.51,0.59)], and a significant negative correlation existed between belief in a just world and maladaptive cognition [*r* = –0.33, 95% CI = (–0.39, –0.27)]; meanwhile, a significant positive correlation existed between internet gaming disorder and maladaptive cognition [*r* = 0.65, 95% CI = (0.59,0.69)]. Moreover, perceptions of school climate [*r* = –0.41, 95% CI = (–0.46, –0.37)] and belief in a just world [*r* = –0.37, 95% CI = (–0.42, –0.32)] were negatively correlated with internet gaming disorder. Although the results of correlation analysis showed the tendency of co-variation among variables, further explorations were needed to clarify the path among variables.

Furthermore, the factor of gender was significantly correlated with the variables of perceptions of school climate, maladaptive cognition, belief in a just world, and internet gaming disorder, and there was also a significant correlation between the place of residence and the perception of school climate among teenage students. So, in follow-up analysis, the factor of gender and place of residence were taken as control variables to reduce the influence of the false effect and undigested effect on the conclusions of this study (Wen, 2017).

### Testing for mediating effect

According to the testing steps of the moderated mediation model proposed by Hayes (2013) and Wen and Ye (2014), we need to test the mediating effect first. Model 4 of the PROCESS macro was used to test the mediating effect of maladaptive cognition between perceptions of school climate and internet gaming disorder. After controlling the variables of gender and place of residence, the model test was run. The results indicated that perceptions of school climate had negative predictive effects on maladaptive cognition (*a* = –0.35, *SE* = 0.03, *p* < 0.001). After including perceptions of school climate and maladaptive cognition into the regression equation at the same time, the negative predictive effects of perceptions of school climate on internet gaming disorder still existed (*c* = –0.10, *SE* = 0.02, *p* < 0.001), and maladaptive cognition had positive predictive effects on internet gaming disorder (*b* = 0.38, *SE* = 0.02, *p* < 0.001). From the percentile bootstrap method test of deviation correction, we inferred that *ab* = –0.13, *boot SE* = 0.01, 95% CI = [–0.16, –0.11], and within the scope of *CI*, 0 was not concluded. So, maladaptive cognition played the mediator role in the relationship between perceptions of school climate and internet gaming disorder, and the ratio of the mediating effect in the total effect was ab/(ab + c) = 57.39%. Hence, Hypothesis 2 was supported.

### Testing for moderated mediation

Then, the moderating effect of belief in a just world among the mediation model should be tested. Due to the hypothesis, we assumed that the belief in a just world had moderating effects on all three paths of the model. Model 59 of the PROCESS macro was used to do the test; three regression equations were established by controlling the variables of gender and age. As shown in Table 4, in Equation 1, perceptions of school climate had negative predictive effects on internet gaming disorder (β = –0.15, *p* < 0.001), and Hypothesis 1 was supported. In Equation 2, perceptions of school climate had negative predictive effects on maladaptive cognition (β = –0.15, *p* < 0.001), and the interaction of perceptions of school climate and belief in a just world also had negative predictive effects on maladaptive cognition (β = –0.08, *p* < 0.01), 95% CI = [–0.12, –0.03]; 0 was not concluded in the interval. So, the belief in a just world played as a moderator in the path of perceptions of school climate to maladaptive cognition. In equation 3, maladaptive cognition had positive predictive effects on internet gaming disorder (β = 0.52, *p* < 0.001), perceptions of school climate had negative predictive effects on internet gaming disorder (β = –0.08, *p* < 0.01), and the interaction of maladaptive cognition and belief in a just world also had positive predictive effects on internet gaming disorder (β = 0.10, *p* < 0.001), 95% CI = [0.06, 0.14]; 0 was not concluded in the interval. So, the belief in a just world played as a moderator in the path of maladaptive cognition to internet gaming disorder. In addition, the interaction of perceptions of school climate and belief in a just world also had negative predictive effects on internet gaming disorder (β = –0.07, *p* < 0.01), 95% CI = [–0.11, –0.02]; 0 was not concluded in the interval. So, the belief in a just world acted as a moderator in the path of perceptions of school climate to internet gaming disorder. Therefore, Hypothesis 3 was supported.

**TABLE 4:** Testing for moderated mediation.

Variables	Equation 1			Equation 2			Equation 3		
	DV:IGD			DV:MC			DV:IGD		
	β	SE	t	β	SE	t	β	SE	t
Gender	0.28***	0.05	6.25	0.26***	0.05	4.69	0.26***	0.04	5.92
Age	–0.05	0.04	–1.14	–0.06	0.05	–1.18	–0.04	0.04	–0.99
PSC	–0.15***	0.02	–6.40	–0.28***	0.03	–8.77	–0.08**	0.03	–2.97
MC							0.52***	0.02	22.35
BJW				–0.16***	0.03	–4.97	–0.14***	0.03	–5.51
SC × BJW				–0.08**	0.02	–3.20	–0.07**	0.02	–3.15
MC × BJW							0.10***	0.02	4.98
*R* ^2^	0.46			0.20			0.50		
F	106.20			59.14			165.21		

DV, dependent variable; PSC, perceptions of school climate; MC, maladaptive cognition; BJW, belief in a just world; IGD, internet gaming disorder; N = 1,164.

**P < 0.01; ***P < 0.001.

**FIGURE 2:**
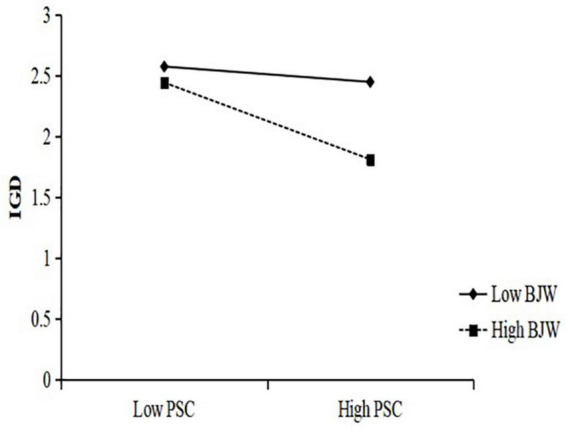
The moderating effect of belief in a just world on perception of school climate to internet gaming disorder.

**FIGURE 3:**
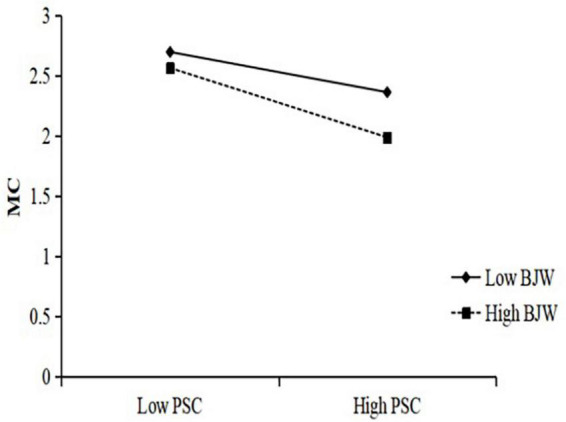
The moderating effect of belief in a just world on perception of school climate to maladaptive cognition.

**FIGURE 4:**
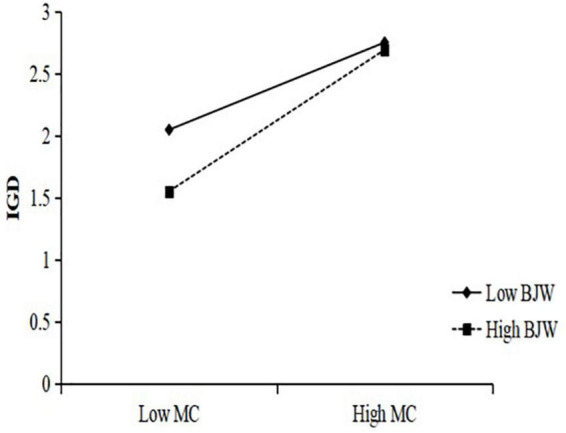
The moderating effect of belief in a just world on maladaptive cognition to internet gaming disorder.

In order to interpret the moderating effect of belief in a just world on the three paths of the mediation model, simple slope analysis were carried out with different levels of belief in a just world (1 SD below the mean and 1 SD above the mean) (Figures 2–4). The results in Figure 2 show that the negative predictive effect of perceptions of school climate on internet gaming disorder was not significant for individuals with low belief in a just world (*B_*simple*_* = –0.15, *p* > 0.05), 95% CI = [–0.30, 0.01]; however, the negative predictive effect of perceptions of school climate on internet gaming disorder was significant for individuals with high belief in a just world (*B*_*simple*_ = –0.73, *p* < 0.001), 95% CI = [–0.87, –0.59]. The results in Figure 3 show that the negative predictive effect of perceptions of school climate on maladaptive cognition was significant for individuals with low belief in a just world (*B_*simple*_* = –0.39, *p* < 0.001), 95% CI = [–0.54, –0.23]; the negative predictive effect of perceptions of school climate on maladaptive cognition was significant for individuals with high belief in a just world, and the effect was greater than that in the group of low belief in a just world (*B*_*simple*_ = –0.67, *p* < 0.001), 95% CI = [–0.80, –0.53]. The results in Figure 4 show that the positive predictive effect of maladaptive cognition on internet gaming disorder was significant for individuals with low belief in a just world (*B*_*simple*_ = 0.44, *p* < 0.001), 95% CI = [0.37, 0.50]; the positive predictive effect of maladaptive cognition on internet gaming disorder was significant for individuals with high belief in a just world, and the effect was greater than that in the group of low belief in a just world (*B_*simple*_* = 0.71, *p* < 0.001), 95% CI = [0.65, 0.76].

Therefore, all three paths of the model that perceptions of school climate affected internet gaming disorder through maladaptive cognition were moderated by belief in a just world.

## Discussion

### Comparison of study variables on demographic variables

Through the comparison of differences, we found that female participants’ perception of school climate and belief in a just world were significantly higher than those of male participants, while the maladaptive cognition and internet game disorder were significantly lower. The result supported the previous studies (Zhang et al., 2014). To schoolgirls, the sense of gain and praises such as a “good girl” can increase their level of confidence and acceptance. So, they prefer to abide by school rules, express social expectation behaviors, and attain greater self-control. Because schoolboys mature later, they are more easily associated with acclimatization to the school culture (Roeser et al., 2000). In the present study, we found that the perception of school climate reported by city students was higher than that of countryside students. This may be related to one’s living environment. Most schools are located in cities; for teenage students who have been accustomed to the city living environment since childhood, the consistency of the environment makes it easier for them to adapt.

### The relationship between perceptions of school climate and internet gaming disorder

In the current study, the significantly negative predictive effect of perceptions of school climate on internet gaming disorder was found among teenage students, that is, teenage students with worse perception of school climate tended to indulge in internet games easily. The result is consistent with previous studies (Bao et al., 2016; Yu et al., 2018), and the stage-environment fit theory is also confirmed. To teenage students, campus is not only the platform for their self-realization but also the platform where social circles satisfy their needs of social communication. So, as the microenvironment, the school plays a decisive role in teenage students’ life. According to the social support theory, the stronger social support network the individuals hold, the more positive behaviors they will choose while dealing with difficulties. The support and encouragement of teachers and students contain enormous energy resources that can satisfy teenage students’ material and emotional needs, stimulate their initiative to seek positive growth, and reduce the internet gaming behavior (Yang et al., 2013; Jia et al., 2017). Moreover, the increase in the sense of gain will promote individuals to involve actively in the process of social communication, and the interaction of interpersonal relationships will help them maintain good perceptions of school climate. According to the social comparison theory, individuals tend to make positive comparisons in an upward environment, and the positive comparisons can arouse individuals’ fighting spirit to seek self-realization (Zhang et al., 2016). On the contrary, if the campus environment and the needs of individual are not compatible, it is difficult to satisfy individuals, negative emotions may be aroused, and then problem behaviors such as aggression, alcoholism, and internet gaming disorder will be initiated easily (Smith, 1981; Eccles and Roeser, 2012; Wang et al., 2017).

### The mediating role of maladaptive cognition

In the current study, we found that maladaptive cognition acted as a mediator in the relationship between perceptions of school climate and internet gaming disorder among teenage students, that is, perceptions of school climate could influence teenage students’ internet gaming disorder through maladaptive cognition. The cognitive–behavioral model of PIU was proved again by the results. As the distal factor, perceptions of school climate affects individuals’ cognition. Moreover, the social cognition of teenagers includes the cognition of themselves and the cognition of others. According to the zone theory of personality development put forward by Erik. H. Erikson, adolescence is the critical period for individuals to establish their ego identity. If individuals feel the possibilities of obtaining ego identity are deprived by the environment, they will try their best to put up a fight (Shaffer and Kipp, 2009). Therefore, teenage students who cannot be satisfied with perceptions of school climate may be dissatisfied with real life. However, the virtualized internet games could provide an outlet for individuals’ emotions, or a platform for exerting their self-value, or even open up a paradise that could replace their social life. At this time, internet games that just cater to the demand of teenagers have planted the seeds of maladaptive cognition in their hearts. Furthermore, as the proximal factor, maladaptive cognition promotes individuals’ behaviors, and the irrational preference for internet games will drive individuals to make top choice under pressure or in the situation of less stimulation, and then the frequencies of unconscious using of online games are increased.

### The moderating role of belief in a just world

Further work showed that belief in a just world could moderate the relationship between perceptions of school climate and internet gaming disorder. Specifically, teenage students with better perception of school climate tended to play less internet games. The effect of low belief in a just world was not significant. On the one hand, teenage students with better perception of school climate prefer to choose more positive prosocial behaviors (Pavin Ivanec et al., 2021). On the other hand, teenage students who have higher belief in a just world are more likely to believe in themselves and others. Thus, they can get more social support and independent opportunities from teachers and classmates to develop the ego identity. Moreover, the satisfaction of self-identity development is the protective factor of teenagers’ internet gaming disorder (Yu et al., 2015). Therefore, teenage students who have good perception of school climate and high belief in a just world have lower tendency of internet gaming disorder.

Belief in a just world could moderate the relationship between perceptions of school climate and maladaptive cognition. Specifically, with a low level of belief in a just world, the better the school climate teenage students perceived, the lower the maladaptive cognition they held; the effect was suitable for teenage students with high levels of belief in a just world, and the connection of the relationship was stronger. Teenage students with better perception of school climate are more likely to get satisfied with their life, and teenage students with higher levels of belief in a just world are more likely to emerge the reciprocal cognition, which can promote the reward behaviors while obtaining social supports (Edlund et al., 2007), and then their own positive behaviors should be promoted. Therefore, teenage students who have good perception of school climate and high belief in a just world have lower tendency of maladaptive cognition.

Belief in a just world could moderate the relationship between maladaptive cognition and internet gaming disorder. Here, we found a very interesting phenomenon that with low levels of belief in a just world, the higher the maladaptive cognition teenage students held, the higher the internet gaming disorder they tended to. The effect was suitable for teenage students with high levels of belief in a just world, and the connection of the relationship was even stronger. After the formation of maladaptive cognition, the belief in a just world played the function of rebuilding the cognition structure. When individuals feel unable to change the reality, they will try to rebuild the justice on their cognitive levels. In this way, they can be satisfied by psychological compensation. The psychological compensation includes constructive compensation and pathological compensation. Individuals can make constructive compensation by rationalization and other methods. While if balance cannot be reached, they can only make pathological compensation by inappropriate ways (such as internet games) to satisfy the temporary needs (Lerner, 1980), or even use the defense strategy of cynicism to escape into the world of internet games. Therefore, the high level of belief in a just world may better motivate teenage students to change their maladaptive cognition. While their resources are not enough to change the reality, the negative behaviors are more likely to be chosen (Shoal et al., 2007; Bao et al., 2016).

From the perspective of culture, individuals are accustomed to think with a holistic cognitive mode and do more external attribution in accordance with Chinese collectivism culture (Liu and Peng, 2012). They pay more attention to the “collective” and hope to integrate into the collective. They help each other, draw ideas and inspiration from the collective, and seek the method for common development. According to previous studies, different from the individualist cultural background, both the personal belief in a just world and general belief in a just world of individuals in the collectivist cultural contribute to generation of positive emotions and behaviors (Wu and Li, 2014). So, we could integrate the belief in a just world into the education of curriculum ideology and politics to establishment the world outlook of teenage students on the one hand, and on the other hand, we could help teenage students establish the personal belief in a just world through their justice experience from the harmonious and fair perceptions of school climate to help them grow healthily.

### Limitations and implications

In this study, there are also several limitations as follows: First, the convenient sampling method might limit the validity of the data, so we can expand the range of sampling for further verification in future research. We can consider adding participants with different cultural and ethnic backgrounds for cross-cultural comparative research to increase the ecological validity of the research. Second, with the cross-sectional design used in this study, the inference of causality among variables might be limited, and the experimental intervention and longitudinal tracking design can be added to clarify the causality further in future research. Third, the self-report method may be affected by the social evaluation effect of the participants, and we can consider adding some measure methods such as others’ evaluation, implicit evaluation, and experimental response to enhance the research validity in future research. Fourth, we can consider adding some variables such as self-control and experience of “flow” to the effect of maladaptive cognition on internet gaming disorder to expand the research angle for further exploration.

## Conclusion

In summary, this study explored the interaction mechanism among perceptions of school climate, maladaptive cognition, belief in a just world, and internet gaming disorder. We found that perceptions of school climate had a predictive effect on internet gaming disorder among teenage students. The path was partially mediated by maladaptive cognition. Moreover, belief in a just world acted as the moderator separately in the path of perceptions of school climate to internet gaming disorder, the path of perceptions of school climate to maladaptive cognition, and the path of maladaptive cognition to internet gaming disorder. Specifically, the effect of perceptions of school climate on internet gaming disorder was significant for teenage students with high levels of belief in a just world. The effect of perceptions of school climate on maladaptive cognition was significant for teenage students with all levels of belief in a just world, and the effect was stronger for those with high levels. Similarly, the effect of maladaptive cognition on internet gaming disorder was significant for teenage students with all levels of belief in a just world, and the effect was stronger for those with high levels. The study enriched the relevant theories of internet gaming disorder on the one hand; on the other hand, it also provided reference value for the intervention of internet gaming disorder among teenage students, such as creating harmonious school climate, integrating the education of firm beliefs into curriculums to help teenage students set up a positive cognitive view, and using psychological consulting to assist the fair education after the formation of maladaptive cognition.

## Data availability statement

The original contributions presented in this study are included in the article/Supplementary material, further inquiries can be directed to the corresponding author.

## Ethics statement

The studies involving human participants were reviewed and approved by the Zhoukou Vocational and Technical College. All participants provided electronic informed consent prior to their participation. Written informed consent to participate under the age of 18 in this study was provided by the participants or their legal guardian/next of kin.

## Author contributions

MZ designed the research and wrote up the manuscript. WZ analyzed the data and wrote up the original draft. YL reviewed literature and revised manuscript. FC collected the data and performed the research. XH designed the structure and developed the methodology. YG reviewed manuscript and supervised the project. All authors contributed to the manuscript revision, read, and approved the submitted version.
